# Special Issue “Innovation and Evidence for Achieving TB Elimination in the Asia-Pacific Region”

**DOI:** 10.3390/tropicalmed6030114

**Published:** 2021-06-28

**Authors:** Philipp du Cros, Hamidah Hussain, Kerri Viney

**Affiliations:** 1 International Development, Burnet Institute, Melbourne 3000, Australia; 2 Interactive Research and Development (IRD), Global IRD, 583 Orchard Road, #06-01 Forum, Singapore 238884, Singapore; hamidah.hussain@ird.global; 3 Department of Global Public Health, Karolinska Institutet, 171 77 Stockholm, Sweden; vineyk@who.int; 4 Research School of Population Health, Australian National University, Canberra 2600, Australia

The World Health Organization’s (WHO) END-TB strategy has set the world on course to climb the highest of medical mountains by 2035, with a targeted peak of reductions in TB deaths by 95%, TB cases by 90%, and no burden of catastrophic expenses on families due to TB [1]. Eliminating TB in the Asia-Pacific region, which has 62% of all estimated TB patients globally, will require innovation, rigorous research, and sustained investment. The Lancet series “How to Eliminate Tuberculosis” outlines an evidence-based approach to ending TB based on four key areas: rethinking data management to target hotspots, active case finding (ACF) and prompt treatment, treating TB infection, and employing a biosocial approach [2]. However, there is still much to learn and improve if this approach is to be effectively implemented. This special issue connects original research and viewpoints on pertinent approaches for improving TB care and prevention in the Asia-Pacific region.

There are multiple components to ACF, and innovation and improvement in all aspects of ACF are required if its contribution to TB elimination is to be maximized. However, evidence that ACF makes a difference at a population level remains limited and the importance of well-conducted evaluations has been highlighted [3]. In this special issue, three studies evaluate ACF interventions conducted in the community. Nguyen et al. reported that community-based, mobile chest X-ray (CXR) screening, regardless of symptoms, followed by Xpert MTB/RIF testing for patients screening positive, had high yields [4]. Half of patients diagnosed had subclinical TB with no or minimal symptoms which is unlikely to be detected with TB symptom screening alone [4]. In a controlled intervention study in Vietnam, Mac et al. reported on an evaluation of community health workers (CHWs) and their ability to refer household contacts (HHCs) and people with symptoms of TB for CXR screening. They reported an 18.3% increase in notifications of all forms of TB [5]. Siahaan et al. evaluated a multicomponent community-based ACF intervention, which included laboratory strengthening and community awareness in two locations in Indonesia. Results from the same intervention differed considerably between the two intervention sites, with a 22% increase in bacteriologically confirmed patients on Nias Island, while there was almost no change in notifications on mainland North Sumatra. They also noted how ACF can place an increased burden on screening and diagnostic cascades, including in the laboratory, which highlights the importance of investments in health system strengthening [6]. 

In this special issue, three approaches to ACF within health facilities are evaluated. Thu et al. presented findings on TB symptom screening performed on adults in a private hospital, followed by CXR screening for those with symptoms. They added a mobile application to reduce dropouts in the cascade of care. This showed high rates of uptake in investigations by persons with possible symptoms of TB and of treatment by those diagnosed with TB [7]. In another study from Vietnam, Vo et al. showed an increase of 8.5% in all forms of TB notifications through subsidized CXR screening and Xpert MTB/RIF testing among private practitioners, with support of improved reporting to the national TB program [8]. In their study, they note that many people treated for TB at private facilities remain unreported, suggesting that further improvements in the intervention process can produce higher yields. Chry et al. reported on a comparison of individual sputum testing compared with a sputum pooling strategy, where both were tested using Xpert MTB/RIF Ultra. The pooling strategy detected 100% of bacteriologically positive people detected by the individual strategy, saving 27% of cartridges and processing time. The authors note that pooling sputum samples for use with Xpert will only have a significant impact on time and costs when programs test a large number of samples in a population with a low yield of TB [9].

HHCs of TB patients are at increased risk of developing TB, especially within the first year after exposure [10]. Four studies in this special issue report on findings from household investigations. Zayar et al. reported on an intervention in which HHCs are screened for diabetes mellitus and active TB. They observed that 14% of HHCs had diabetes mellitus and 5% had active TB, and there was no difference in TB prevalence among HHCs based on diabetes status of the index TB case [11]. Two studies reported on evaluations of HHCs among multi-drug resistant (MDR) TB index patients in Myanmar. Phyo et al. reported on the diagnostic cascade of care and noted significant disparities in reaching health care facilities and in investigations for persons with possible symptoms of TB. Kyaw et al. reported a yield of active TB of 3.9% among HHCs of MDR-TB index patients, with the highest yield of 10% among children under 5 years old. However, only a minority of TB patients diagnosed had rifampicin resistant (RR) TB [12,13]. Finally, Paryani et al. reported an incidence rate of TB of 2072/100,000 person years in the HHCs of index patients with pre-extensively drug resistant (pre-XDR) and XDR-TB following initial screening, which shows the importance of ongoing follow up and preventive treatment among HHCs [14].

Das et al. reported on a before and after intervention study in a conflict-affected area in India, with the introduction of Xpert MTB/RIF and patient follow-up supported by community health workers, implemented in mobile clinics [15]. This showed an increase in bacteriological confirmation and a reduction in pre-treatment loss to follow up. Anh et al. reported on high treatment success rates with levofloxacin and an injectable containing short treatment regimen for patients with RR-TB in Vietnam [16]. Lohiya et al. described the outcomes for patients with drug resistant (DR)-extrapulmonary TB in a retrospective analysis in three DR-TB treatment centers in Delhi, India, showing that one-third of patients had unfavorable treatment outcomes [17].

Finally, three perspective pieces describe some of the broader aspects required to improve TB care. Creswell et al. described the experience of the STOP TB Partnership’s TB REACH initiative. This novel mechanism funds projects that develop, evaluate, and expand innovative approaches to improve TB case finding, treatment, biosocial approaches, and prevention [18]. Satyanarayana et al. described the opportunities to address the major socioeconomic determinants of TB through the lens of the Sustainable Development Goals [19]. Harries et al. examined the important role of TB preventive treatment in achieving TB elimination. They describe how research can improve the implementation of TB preventive treatment, highlighting the need for innovation, improved diagnostic tools, better biomarkers for the progression of TB infection to TB disease, and improved adherence monitoring tools [20].

While the challenges for TB elimination remain large, there is still cause for optimism, with new evidence on screening and ACF, improved all-oral MDR-TB treatment regimens, an increasing array of novel TB diagnostics, and shorter treatment regimens for TB preventive treatment. While progress has been made, the COVID-19 pandemic has highlighted how fragile progress on TB can be, with 1.4 million fewer TB patients diagnosed in 2020 [21]. With increased TB transmission and mortality due to TB predicted, the pandemic may set back progress by a decade [21]. Achieving TB elimination will require greater investments in screening, diagnosis, treatment, and prevention as well as major socioeconomic change. It will also require increased research, innovation, and evaluation in order to find the most appropriate people-centered models of care for all countries and contexts. 
